# Does clinical T1N0 GGN really require checking for distant metastasis during initial staging for lung cancer?

**DOI:** 10.1186/s40644-024-00714-7

**Published:** 2024-06-03

**Authors:** Kazuhiro Imai, Nobuyasu Kurihara, Motoko Konno, Naoko Mori, Shinogu Takashima, Shoji Kuriyama, Ryo Demura, Haruka Suzuki, Yuzu Harata, Tatsuki Fujibayashi, Sumire Shibano, Akiyuki Wakita, Yushi Nagaki, Yusuke Sato, Kyoko Nomura, Yoshihiro Minamiya

**Affiliations:** 1 Department of Thoracic Surgery, Akita University Graduate School of Medicine, 1-1-1 Hondo, Akita, 010-8543 Japan; 2 Department of Radiology, Akita University Graduate School of Medicine, Akita, Japan; 3 Department of Health Environmental Science and Public Health, Akita University Graduate School of Medicine, Akita, Japan

**Keywords:** Lung neoplasms, Diagnostic screening programs, Neoplasm staging, Incidental findings

## Abstract

**Background:**

Accurate clinical staging is crucial for selection of optimal oncological treatment strategies in non-small cell lung cancer (NSCLC). Although brain MRI, bone scintigraphy and whole-body PET/CT play important roles in detecting distant metastases, there is a lack of evidence regarding the indication for metastatic staging in early NSCLCs, especially ground-grass nodules (GGNs). Our aim was to determine whether checking for distant metastasis is required in cases of clinical T1N0 GGN.

**Methods:**

This was a retrospective study of initial staging using imaging tests in patients who had undergone complete surgical R0 resection for clinical T1N0 Stage IA NSCLC.

**Results:**

A total of 273 patients with cT1N0 GGNs (*n* = 183) or cT1N0 solid tumors (STs, *n* = 90) were deemed eligible. No cases of distant metastasis were detected on initial routine imaging evaluations. Among all cT1N0M0 cases, there were 191 incidental findings on various modalities (128 in the GGN). Most frequently detected on brain MRI was cerebral leukoaraiosis, which was found in 98/273 (35.9%) patients, while cerebral infarction was detected in 12/273 (4.4%) patients. Treatable neoplasms, including brain meningioma and thyroid, gastric, renal and colon cancers were also detected on PET/CT (and/or MRI). Among those, 19 patients were diagnosed with a treatable disease, including other-site cancers curable with surgery.

**Conclusions:**

Extensive staging (MRI, scintigraphy, PET/CT etc.) for distant metastasis is not required for patients diagnosed with clinical T1N0 GGNs, though various imaging modalities revealed the presence of adventitious diseases with the potential to increase surgical risks, lead to separate management, and worsen patient outcomes, especially in elderly patients. If clinically feasible, it could be considered to complement staging with whole-body procedures including PET/CT.

**Supplementary Information:**

The online version contains supplementary material available at 10.1186/s40644-024-00714-7.

## Background

In cases of non-small cell lung cancer (NSCLC), accurate clinical staging is crucial for selection of optimal oncological treatment strategies and surgical procedures. For example, for early-stage NSCLC, including ground-glass nodule (GGN) adenocarcinomas, which grow very slowly (volume doubling time > 700 days) [1], surgical resection is associated with favorable prognoses [2, 3].

MRI, bone scintigraphy (BS), and whole-body fluorine 18 (^18^F) fluorodeoxyglucose (FDG) PET/CT play important roles in detecting distant metastases in NSCLCs at presentation. For preoperative evaluation, PET/CT is recommended over surgical staging for NSCLC patients with abnormal mediastinal/hilar lymph nodes on CT and is probably even more useful for metastatic staging [3–6]. One important question is whether it is necessary to evaluate the possible existence of brain metastases with brain MRI and/or bone metastases with BS. There is some controversy between existing guidelines, especially for early-stage NSCLC, as the detection rate of distant metastases is very low [3, 4, 7–11]. In general, an initial routine brain MRI is unnecessary for patients with GGNs and subsolid nodules [2] because preoperative staging does not have prognostic benefit for survival [12, 13].

Incidental findings believed to be clinically important are reported in 5–20% of CT lung cancer screening alone [11]. These are also frequently seen on various staging imaging modalities and raise additional concern for invasive procedures in NSCLC management. As demonstrated by the inconsistent guidelines [3, 4, 14] and variability in daily clinical practice, there is a lack of evidence regarding the indication for metastatic staging at initial presentation of early-stage cT1N0 NSCLC. In the present study, we focused on the preoperative management of resectable cT1N0 GGNs. Our aim was to confirm whether clinical T1N0 GGNs really require checking for distant metastasis during initial lung cancer staging.

## Methods

### Ethical requirements

All experimental protocols were approved by the institutional review board at Akita University Hospital (approval number: 2679). All data were collected under this IRB Protocol, which allows collection of medical record with consent or waiver of consent when no personalized health information is required, as was the case in this study. An opt-out approach was used for this retrospective study.

### Patients

**Fig. 1:**
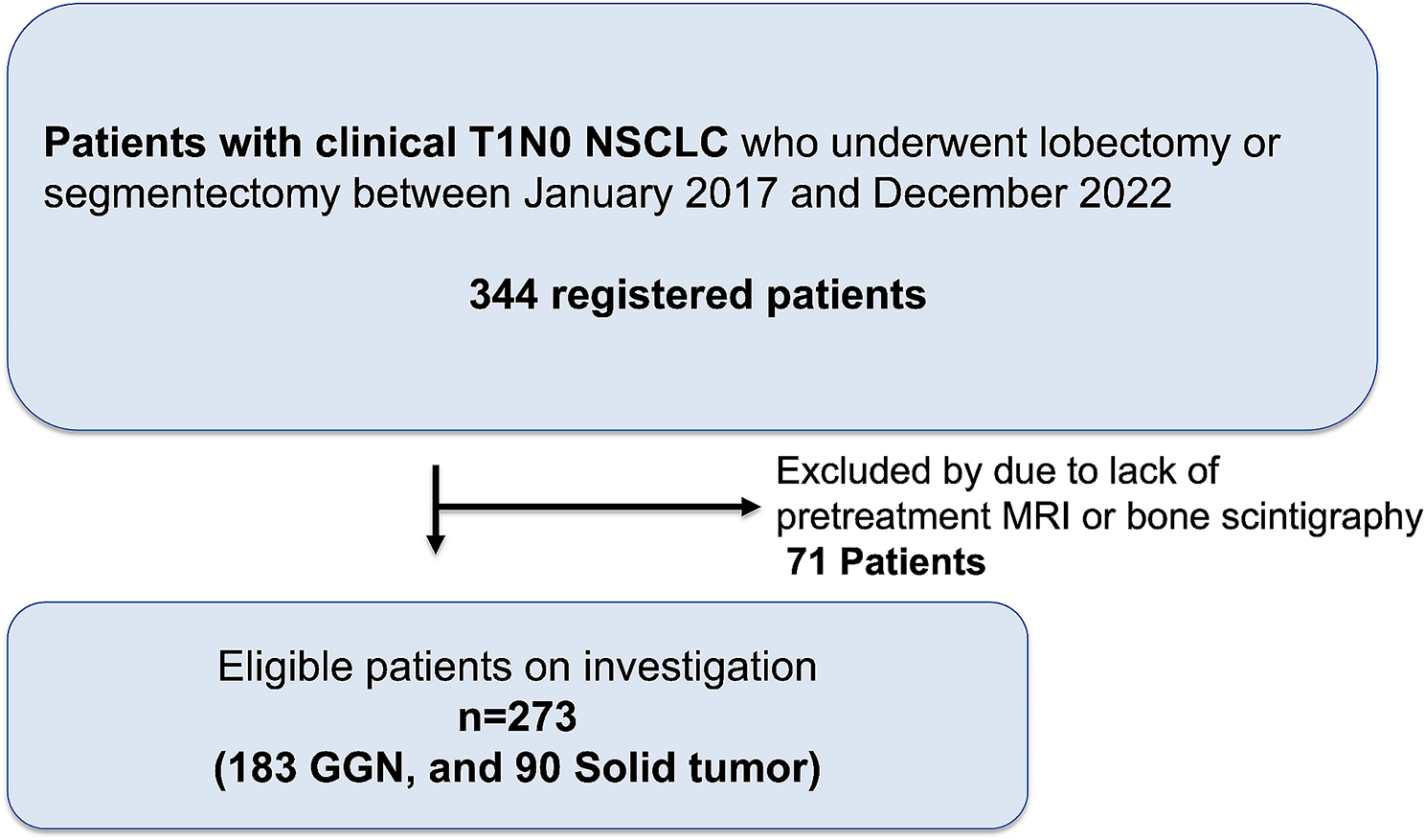
Flow chart illustrating the subject enrollment protocol

This was a single-center study of initial staging using imaging tests in patients who had undergone complete surgical resection (R0 resection) for clinical T1N0 Stage IAs NSCLC. The medical records of 344 clinical T1N0 NSCLC patients who underwent lobectomy or segmentectomy at our institute between January 2017 and December 2022 were retrospectively reviewed. Of those, 71 cT1N0 patients who did not receive pretreatment screening brain MRI and/or BS were excluded. The remaining 273 participants, who had cT1N0 GGNs, including part-solid nodules (GGN group, *n* = 183), or cT1N0 solid tumors (ST group, *n* = 90) were deemed eligible for investigation and comparison. The patients’ characteristics are listed in Table 1. A diagram of the process by which cases were selected for study is shown in Fig. 1.

**Table 1:** Characteristics of patients with clinical T1N0 non-small cell lung cancer

	GGN (*n* = 183)	Solid tumor (*n* = 90)	*p*-value
Age, median (range)	72 (39–86)	72 (46–85)	0.5641
Sex male/female, n	82/101	52/38	0.534
Brinkman index, average	327.5	706.5	< 0.0001*
**Clinical staging by pre-op CT**			
cTis/T1mi/T1a/T1b/T1c	23/28/51/65/15	0/0/3/52/35	< 0.0001*
Tumor size, avg. (range), mm	21.5 (6–56)	19.1 (9–29)	0.0074*
Maximum size of a solid lesion, avg. (range), mm	9.7 (0–28)	18.9 (9–29)	< 0.0001*
**Pre-op distant metastasis**	0	0	
**Histology**, n			
AIS	28	2	
Adenocarcinoma	155	62	
Squamous cell carcinoma	0	25	
others	0	1	
**Surgery**, n			
Lobectomy/*Segmentectomy*	106/*77*	69/*21*	
30 days mortality	0%	0%	
**Pathological staging**			
pTis/T1mi/T1a/T1b/T1c/T2a/T3	36/56/41/33/12/5/0	2/3/12/37/23/11/0/2	< 0.0001*
pN0/1/2	183/0/0	79/**7**/**4**	< 0.0001*
**Incidental findings by pre-op tests**, n (%)			0.8962
+	108 (59.02%)	52 (57.8%)	
−	75 (40.98%)	38 (42.2%)	

GGN, ground-grass nodule

### Preoperative imaging for determining clinical staging

In addition to chest CT, all patients underwent brain MRI and planar BS and/or whole-body ^18^F-FDG PET/CT as routine procedures within 3 months before surgery.

Revolution CT (GE Healthcare) or other model was used for preoperative testing. Typical scan parameters were 60 keV and auto-mA. The scan area always included the chest, whereas inclusion of the abdomen to the pelvic region varied by case. The scans included both contrast-unenhanced and contrast-enhanced CT (pulmonary arterial and venous phase and equilibrium phase). Five-mm- and 0.625-mm-thick axial and 2mmthick coronal and sagittal sections were reconstructed. High-resolution computed tomography reconstructed 1.25-mm axial sections with a field of view of 180 mm.

Contrast-enhanced brain MRI to search for brain metastases was performed using a 3T system (Vantage Cencurian, Canon, or Discovery MR750, GE Healthcare) or other systems using a standard head coil. Examinations included axial T2-weighted images with turbo spin-echo, axial fluid-attenuated inversion-recovery images, axial contrast-unenhanced T1-weighted images with spin-echo (SE), axial and coronal contrast-enhanced T1-weighted images with SE and a contrast-enhanced three-dimensional gradient-echo pulse sequence. Contrast-enhanced sequences were obtained at least 5 min after intravenous injection of the contrast agent. MR angiography of the head and neck to detect arterial stenosis was added at the discretion of the radiologist.

BS was performed using a dual head gamma camera (Symbia Evo or Symbia E Dual Head System, Siemens Healthcare GmbH) with low-energy and high-resolution collimator. 900–1000 MBq of 99 m-Technetium (^99m^Tc)-Methylene diphosphonate (MDP) (PDRadiopharma Inc.) or ^99m^Tc-Hydroxy methylene diphosphonate (HMDP) (Nihon Medi-physics Co., Ltd.) was injected intravenously. Data acquisition was started after 3–4 h. The imaging parameters were a matrix size of 1024 × 256 and a bed speed of 9–12 cm/min. A subsequent tomography (single-photon emission computed tomography) was performed as needed.

PET/CT image of ^18^F-FDG was obtained using Biograph Vision 600 (Siemens Healthcare GmbH) or Discovery ST Elite 16 (GE Healthcare). 3.7 MBq/kg of ^18^F-FDG (Nihon Medi-physics Co., Ltd.) was injected venously and data acquisition began 60 min later. The PET imaging range was from the top of the head to the proximal 1/3 of the femur. The collection method was either whole-body dynamic imaging at 3 mm/sec for 4 times and additive reconstruction, or 3 min/bed (8–9 beds). A diagnostic CT scan for fusion was obtained using a standard protocol without intravenous contrast (120 kV; auto mA range, 20–666 mA; thickness, 3–3.75 mm; pitch, 1.2–1.75).

Co-registered images were displayed and analyzed using a high-speed 3D-image analysis system that enabled visualization of medical images in 3D for diagnosis and surgical simulation (SYNAPSE VINCENT, Fujifilm Corporation, Tokyo, Japan).

All patients had CT, brain MRI, and BS. 126 of 183 patients (68.9%) in the GGN group and 68 of 90 patients (75.6%) in the ST group had PET/CT. For evaluations using CT, MRI, BS and/or PET/CT, tumor size, lymph nodes, distant metastasis and staging were classified based on their location (i.e., mediastinal or hilar) and the 8th edition of the Union Internationale Contre le Cancer (UICC)-TNM staging system [15]. Board-certified thoracic surgeons (KI, NK, ST, SK, RD, HS, YH, TF, SS, AW, YN, YS, and YM) and radiologists (MK, NM and colleagues) evaluated the results of these preoperative tests for clinical staging (and detection of other diseases). Incidental findings are defined as incidentally discovered masses or lesions detected for an unrelated reason [16, 17].

### Surgical procedure and follow-up

All patients received standard pre- and intraoperative care, and radical segmentectomy/lobectomy plus systemic node dissection. Pathological staging was also based on the 8th edition of the UICC-TNM classification [15]. Although the follow-up schedule after surgery varied, it usually entailed a chest CT every 3–6 months and others every 6–12 months for the first 2 years. If recurrence was suspected, the follow up schedule was tightened.

### Statistical analysis

Clinical characteristics were statistically compared between the GGN and ST groups. Continuous variables were investigated using unpaired t tests or the Wilcoxon/Kruskal-Wallis test, while categorical variables were investigated using the Chi-squared test with continuity correction or Fisher’s exact test, as appropriate. 5-year overall survival (OS), relapse-free survival (RFS) and disease-free survival (DFS), and compared between the GGN and ST groups using the log-rank test. The RFS was calculated as the time from surgery of disease to any event, irrespective of cause, except for any second primary cancers. The DFS was the time from random assignment to cancer recurrence or death from any cause. Patients known to be alive or lost to follow-up on the date of last contact were treated as censored. Univariate and multivariate logistic regression analyses assessed the relationship between key variables and incidental findings. We calculated odds ratios along with 95% Confidence Interval (95%CI). All statistical analyses were performed using JMP IN 17.0.0 software (SAS Institute, Cary, NC, USA). P-values were 2-sided and considered significant if less than 0.05.

## Results

**Fig. 2:**
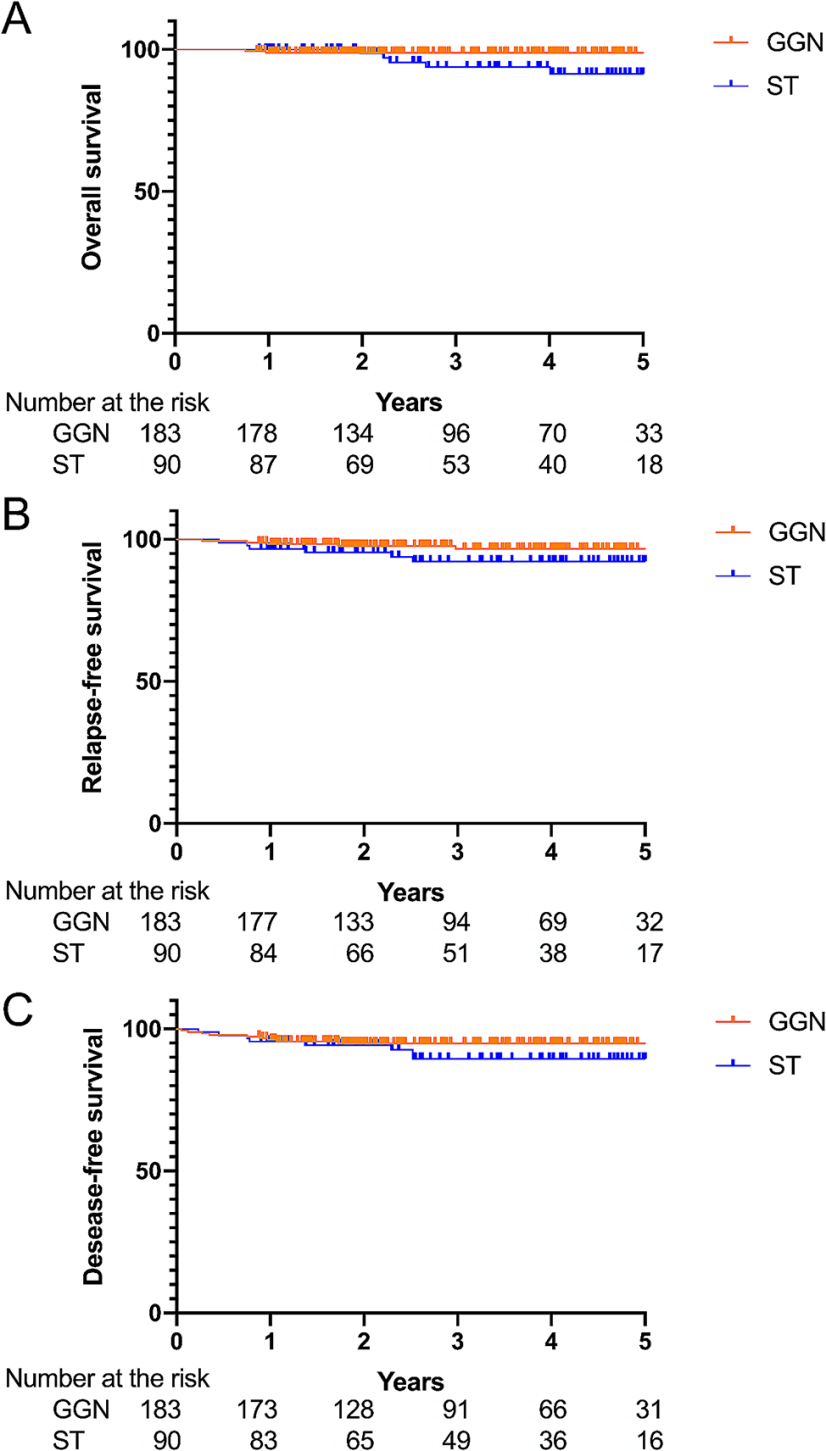
Kaplan-Meier curves comparing 5-year overall survival, relapse-free survival, and disease-free survival between patients with cT1N0 ground-grass nodules (GGNs) and solid tumors (STs). Only overall survival differed between the GGN and ST groups (*p* = 0.0382)

The clinical characteristics of the 183 patients with GGNs and 90 with STs are compared in Table 1. The 5-year OS, RFS and DFS among patients with cT1N0 GGNs were 98.9%, 96.7% and 94.9%, respectively, and 91.4%, 92.2% and 89.5% among patients with cT1N0 STs. Kaplan-Meier analysis showed that only OS differed between the GGN and ST groups (HR = 4.799, 95% CI: 1.007–22.86, *p* = 0.0382, Fig. 2). No cases of distant metastasis were found during the initial routine imaging evaluations. During the median follow-up of 3.36 years (interquartile range, 0.75–7.5 years), 2.7% (5/183) of GGN patients and 6.7% (6/90) of ST patients experienced recurrence of their lung cancer (*p* = 0.1865). There was no significant difference in the percentages of incidental findings during preoperative tests between the two groups (*p* = 0.8962).

Table 2A summarizes the incidences of adventitious diseases detected during preoperative testing. Among all 273 study participants, MRI revealed cerebral leukoaraiosis to be the most frequently detected incidental diagnosis, occurring in 98 (35.9%) patients, while cerebral infarction was found in 12 (4.4%) patients. In both GGN and ST patients, PETCT and brain MRI also revealed a variety of treatable neoplasms, including brain meningioma, parotid gland tumor (Warthin tumor), thyroid benign tumor/cancer, femoral nerve schwannoma, gastric cancer, renal cancer and colon cancer. Notably, four cases of carotid artery arteriosclerotic disease, putting the patient at high risk for ischemic stroke or transient ischemic attack (TIA), were first diagnosed only on MRI. In addition, there were 47 incidental findings on pre-testing CT (Table 2B). Of those, cases of breast cancer, gallbladder cancer and pancreatic intraductal papillary mucinous neoplasm were found and diagnosed as treatable malignant neoplasms.

**Table 2A:** Adventitious diseases detected on preoperative bone scintigraphy, brain MRI and PET/CT when checking for distant metastasis during initial staging

	GGN (*n* = 183)	Solid tumor (*n* = 90)	All T1 patients (*n* = 273)
**Low clinical significance**			
Cerebral leukoaraiosis	67*	31*	98 (35.9%)
Cavernous malformation	2	0	2
Subdural hygroma	0	1	1
Sinusitis	3	0	3
Chronic thyroiditis	0	2	2
Spinal disc hernia	1	0	1
Gastro esophageal reflux disease	0	1*	1
Bone marrow hyperplasia	1	0	1
**High clinical significance**			
Cerebral infarction	8*	4*	12 (4.39%)
Cerebral hemorrhage	1	0	1
Carotid artery stenosis	2**	1	3
Carotid artery aneurysm	0	1	1
**Benign tumor**			
Brain lipoma	1	0	1
Parotid gland tumor (Warthin tumor)	1	1	2
Thyroid benign tumor	3	0	3
Femoral nerve schwannoma	1	0	1
Adrenal adenoma	1	1	2
Spleen hemangioma	1	0	1
Ovarian cyst	1	0	1
**Malignant potential tumor**			
Meningioma	**1**	**1**	**2**
Thyroid cancer	0	**1**	**1**
Gastric cancer	**1**	**1**	**2**
Renal cancer	**1**	0	**1**
Colon cancer	0	**1**	1
Total	97	47	**144**

*Finding was counted even when one patient had multiple diseases, **including vertebral artery occlusion

**Table 2B:** Other adventitiously diseases detected on preoperative CT. All patients received contrast-enhanced chest CT to the inferior margin of the liver for lung cancer staging

	GGN (*n* = 183)	Solid tumor (*n* = 90)	All T1 patients (*n* = 273)
Abdominal CT to Pelvis, pt (%)	147 (80.3%)	72 (80%)	219 (80.2%)
**Low clinical significance**			
Shoulder periarthritis	0	1	1
Hiatal hernia	1	0	1
Abdominal aortic mural thrombus	0	1	1
Chronic pancreatitis	1	0	1
Splenic artery aneurysm	1	0	1
Renal stone	1*	1	2
Renal artery aneurysm	1	0	1
Colonic diverticulum	2*	0	2
Prostatic hypertrophy	1	0	1
Inguinal hernia	1	0	1
**High clinical significance**			
Pulmonary embolism	0	1*	1
Thoracic compression fracture	1	0	1
Iliac artery stenosis	1	1	2
Hydronephrosis	1	1	2
**Benign tumor**			
Thyroid benign tumor	4*	1*	5
Plummer disease	0	1	1
Breast fibroadenoma	1	0	1
Gastric submucosal tumor	1	0	1
Liver cyst	0	1*	1
Gallbladder adenomyosis	0	1	1
Splenic hemangioma	0	1	1
Renal cyst (including hemorrhagic)	1	1	2
Adrenal adenoma	2*	0	2
Abdominal schwannoma	1	0	1
Ovarian cyst	1	1	2
Ovarian teratoma	0	1	1
Uterine fibroid	3*	2	5
Testicular tumor	2*	0	2
**Malignant potential tumor**			
Breast cancer	1	0	1
Gallbladder cancer	1	0	1
IPMN	1	0	1
Total	31	16	**47**

*Finding was counted even when one patient had multiple diseases, IPMN; intraductal papillary mucinous neoplasm

**Fig. 3:**
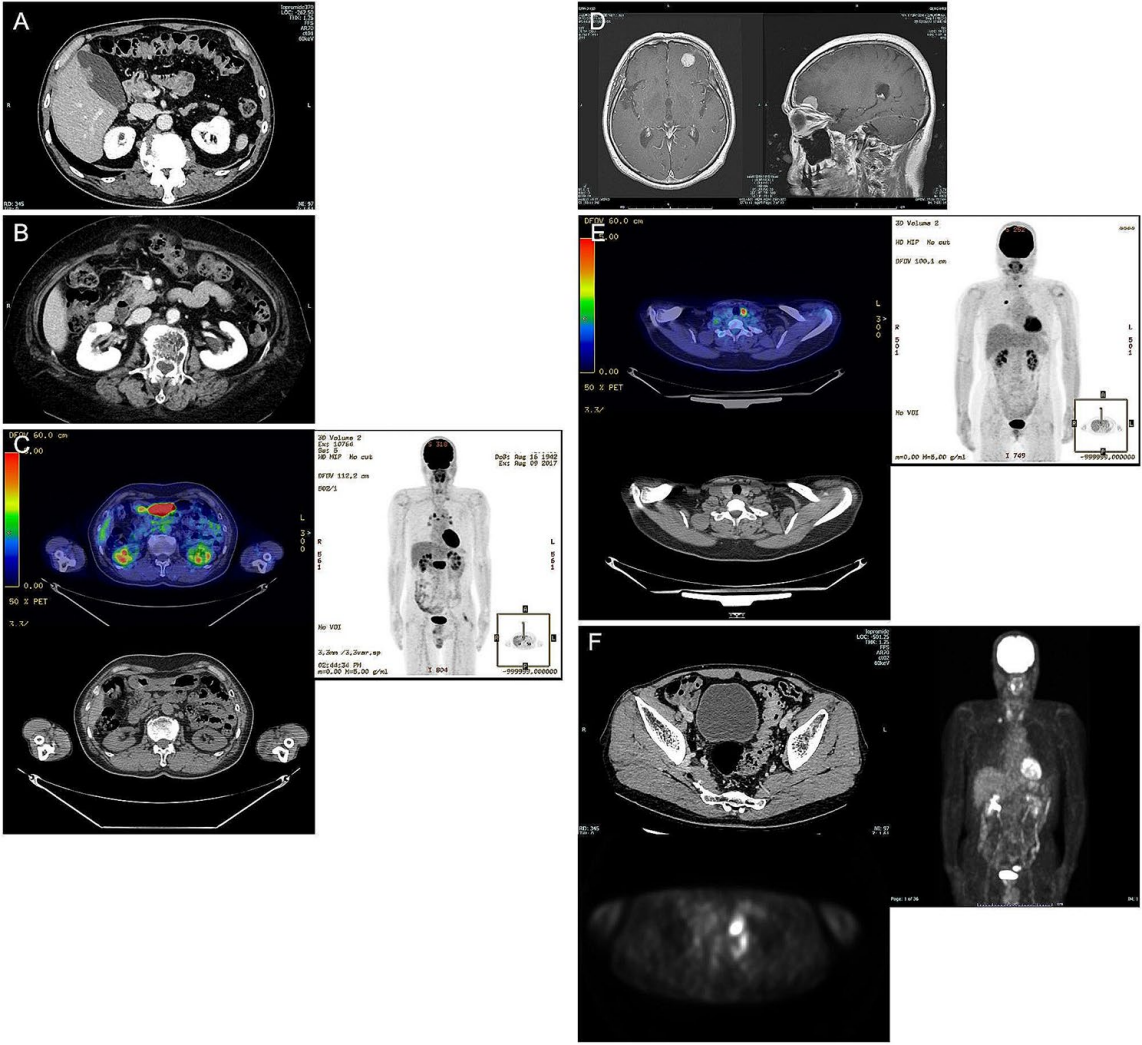
Incidental findings of treatable malignant potential tumor during preoperative imaging **(A) Case 4** (71-year-old male with bilateral GGNs in right S1 and left S1 + 2): Gallbladder cancer was detected on contrast-enhanced (CE)-CT. CT revealed 18 mm pedunculated tumor without invasion into the liver parenchyma. Case 4 received extended cholecystectomy before pulmonary resection. **(B) Case 7** (86-year-old female with right S6 GGN): Renal cancer was detected on CE-CT and PET/CT. The 10 mm nodule showed hyperenhancement in the arterial phase and washout in the equilibrium phase of CE-CT. Case 7 was followed up with CT after pulmonary resection. **(C) Case 8** (75-year-old male with right S7 GGN): Gastric cancer was detected on PET/CT. PET/CT showed anterior gastric wall thickening and high maximum standardized uptake value (SUV_max_ 27.9) in the pyloric end of stomach. After pulmonary resection, Case 8 received distal gastrectomy with D2 lymph node dissection and the final pathological stage was IIIB. **(D) Case 9** (77-year-old female with left S1 + 2 GGN and left breast cancer 5.5 cm, pT1cN0): Meningioma was detected on brain MRI. The enhancing brain mass adjacent to the anterior cranial base was 20 mm, with edematous. Case 9 was followed up with CT after pulmonary resection. Case 9 had no tumor-related symptoms. **(E) Case 17** (56-year-old female with right S1 solid adenocarcinoma): Thyroid papillary carcinoma was detected on PET/CT. The low-density nodule showed SUV_max_ 7.0. Case 17 received thyroidectomy after pulmonary resection **(F) Case 18** (68-year-old male with right S1 solid adenocarcinoma): Sigmoid colon cancer was detected on CE-CT and PET/CT (conducted at another hospital). PET/CT revealed sigmoid colonic wall thickening and high FDG uptake. Case 18 received laparoscopic sigmoidectomy with D2 lymph node dissection before pulmonary surgery

Table 3 summarizes the patients with incidental findings of treatable diseases detected during preoperative testing. In total, 19 patients were diagnosed with a treatable disease. In Cases 3 and 6, carotid stenosis with/without vertebral artery occlusion was found on MRI in patients with GGN; they received HMG-CoA reductase inhibitors before surgery. In Case 10, an 80-year-old female patient with ST adenocarcinoma was diagnosed with cerebral leukoaraiosis on initial brain MRI, and cerebral infarction suddenly developed as a foreseeable complication on post-operative day 3; she was treated with mechanical thrombectomy using a stent retriever and improved with no sequelae. In Case 4, gallbladder cancer was suspected/detected on CT in a patient with GGN (Fig. 3A); he received extended cholecystectomy before pulmonary resection. In Case 7, renal cancer was suspected based on ^18^F-FDG uptake on PET/CT in a patient with GGN (Fig. 3B); she was followed up with CT after pulmonary resection because she was more than 85 years old. In Cases 8 and 11, patients with GGN (Fig. 3C) and ST, respectively, were suspected of having gastric cancer based on ^18^F-FDG uptake on PET/CT; they received radical gastrectomy. In Case 9, meningioma (Fig. 3D, follow-up) was detected on brain MRI and breast cancer on chest CT in a patient with GGN; she received a partial mastectomy plus sentinel lymph node dissection concurrently with pulmonary resection. The final pathological diagnosis of breast cancer was invasive ductal carcinoma, pT1cN0. In Case 17, thyroid cancer (papillary carcinoma) was detected in a patient with ST (Fig. 3E) and surgically-resected. In Case 18, sigmoid colon cancer (Fig. 3F) was detected on contrast-enhanced CT and PET/CT at the same time as the lung cancer in a patient with ST; he received a laparoscopic sigmoidectomy (type 0-I, 20 mm, tub1, pT1sN0, pStage 0, CurA) before pulmonary surgery. In Case 19, a cerebral infarction was diagnosed from preoperative MRI in a patient with ST; he received anticoagulant medicines after surgery. Figure S1 shows images of the incidental findings in patients considered high surgical risk candidates.

**Table 3:** Summary of patients with treatable adventitious tumors (or diseases) detected during preoperative testing

Case	Age	Sex	Primary	cT	pT, pStage	Device	Found Disease	Treatment
1	70	M	GGN (adeno)	1b	2a, IB	PET	**Adrenal adenoma**	Follow-up
2	83	M	GGN (adeno)	1a	1c, IA3	MRI	**Parotid gland tumor (Warthin tumor)**	Follow-up
3	67	M	GGN (adeno)	1mi	1mi, IA1	MRI	**Vertebral artery occlusion**	HMG-CoA Reductase Inhibitors
4	71	M	GGN (adeno)	1a	is, 0	CE-CT	**Gallbladder cancer**	*Extended cholecystectomy*
5	56	M	GGN (adeno)	1b	1b, IA2	PET	**Femoral nerve schwannoma**	Follow-up
6	78	M	GGN (adeno)	1a	is, 0	MRI	**Carotid stenosis**	HMG-CoA Reductase Inhibitors
7	86	F	GGN (adeno)	1b	1a, IA1	CE-CT	**Renal cancer**	Follow-up
8	75	M	GGN (adeno)	1b	1b, IA2	PET	**Gastric cancer**	*Distal gastrectomy*
9	77	F	GGN (adeno)	1a	1b, IA2	MRI	**Meningioma / Breast cancer**	Follow-up / Bp + SLND
10	80	F	adeno	1b	1b, IA2	MRI	Cerebral leukoaraiosis-infarction*	*Catheter thrombectomy*
11	77	M	adeno	1b	1b, IA2	PET	**Gastric cancer**	*Laparoscopic total gastrectomy*
12	76	M	sq	1b	1a, IA1	PET	**Adrenal adenoma**	Follow-up
13	73	M	sq	1b	1b, IA2	MRI	**Carotid stenosis**	Follow-up
14	72	M	sq	1c	1c, IA3	MRI	**Carotid aneurysm**	Follow-up
15	85	F	AIS	1b	is, 0	MRI	**Meningioma**	Follow-up
16	66	M	adeno	1b	1b, IA2	PET	**Parotid gland tumor (Warthin tumor)**	Follow-up
17	56	F	adeno	1b	1a, IA1	PET	**Thyroid papillary carcinoma**	*Thyroidectomy*
18	68	M	adeno	1c	2a, IB	CE-CT, PET	**Colon cancer**	*Sigmoidectomy*
19	68	M	sq	1c	2a, IB	MRI	**Cerebral infarction**	*Anticoagulant drugs*

adeno, adenocarcinoma; sq, squamous cell carcinoma; CE, contrast-enhanced; Bt, partial breast mastectomy; SLND, sentinel lymph node dissection

* acute cerebral infarction developed on post-operative day 3

Table 4 shows the univariate and multivariate analyses with logistic regression analyses. Age, gender, Brinkman index, and ST (or GGN) were used as predictors. The analyses revealed that only age 75 years and older was associated with incidental findings.

**Table 4:** Influence of patient characteristics on rate of incidental findings

	Univariate analysis			Multivariate analysis		
IFs by MRI, BS, and PET/CT	Odds ratio	95% CI	*p*-value	Odds ratio	95% CI	*p*-value
Age: ≥75 versus < 75	**2.703**	1.615–4.522	0.0002*	**2.656**	1.582–4.461	0.0002*
Gender: male versus female	1.447	0.898–2.331	0.1287	1.351	0.747–2.442	0.3194
Brinkman index	2.131	0.538–8.445	0.2817	1.170	0.202–6.769	0.8607
GGN versus Solid tumor	0.926	0.559–1.535	0.7663	0.839	0.488–1.439	0.5232
IFs by all modalities (preoperative CT, + MRI, BS, and PET/CT)						
Age: ≥75 versus < 75	**2.606**	1.529–4.440	0.0004*	**2.575**	1.508–4.401	0.0005*
Gender: male versus female	1.233	0.761–1.998	0.3946	1.117	0.614–2.032	0.7162
Brinkman index	1.786	0.437–7.301	0.4196	1.292	0.213–7.817	0.7806
GGN versus Solid tumor	0.950	0.569–1.585	0.8451	0.877	0.509–1.513	0.6378

* Significant difference

IFs, incidental findings; GGN, ground-grass nodule; BS, bone scintigraphy; CI: confidence interval

## Discussion

In the present study, we found that in clinical T1N0 GGN patients, checking for distant metastasis is not required for initial staging, but preoperative imaging tests should be conducted because incidental findings of other diseases, including ischemic diseases, peripheral arterial disease, treatable benign/malignant neoplasms, were frequently seen on various modalities. These incidental findings have the potential to increase surgical risks and worsen patient outcomes.

PET/CT has improved diagnostic accuracy and influenced initial cancer staging. Therefore, surgical resection is generally recommended for patients with a non-centrally located resectable lung cancer and an absence of nodal metastasis on PET/CT images [3]. PET/CT better identifies extra-thoracic metastases, sparing some from stage-inappropriate surgery. Notably, segmentectomy for cStage IA GGNs and lung adenocarcinomas provides excellent long-term survival to selected patients with disease that meets the N0 criteria, such as a tumor maximum standardized uptake value of < 1.5 on ^18^F-FDG PET/CT [18]. In a meta-analysis evaluating the accuracy of PET/CT for diagnosis of distant metastasis in lung cancer [19], the sensitivity and specificity of PET/CT were 0.92 and 0.97, and PET/CT had excellent diagnostic performance for metastasis (M) staging. On the other hand, PET/CT is not routinely necessary for staging pure GGNs because abnormal ^18^F-FDG uptake by both the lymph nodes and other organs is almost never found [2] and actual lymph/distant metastasis is extremely rare (0.1% N1 node metastasis and no N2 disease or distant metastasis was found) [20–22]. Indeed neither lymph node metastasis nor distant metastasis was found in the present or other studies [2, 22], and PET/CT provided no additional information with regard to managing GGNs [23]. On the other hand, 5.5% of clinical T1N0 GGN patients had incidental findings of other diseases (10/183 GGN patients) on PET/CT in the present study.

Although the central nervous system is a frequent extra-thoracic site from lung cancer metastases and about 10-20% of NSCLC patients have already developed brain metastases by initial staging, most guidelines recommend brain MRI for cStage II to IV and discourage it for cStage I, especially IA [3, 4, 12, 24–26]. In cStage IA disease, the diagnostic yield of staging brain MRI was 0.3% [14]. The particularly low diagnostic yield provides evidence that there is no need for staging brain MRI in cStage IA disease, including GGNs, whereas staging brain MRI should be considered in cStage IB disease (the yield was 3.8%) or epidermal growth factor receptor (EGFR) mutation-positive adenocarcinoma (17.5%) [14]. Whether MRI is cost-effective for patients with cStage IA disease is unclear, given the very low brain metastasis rate. However, to be safe, all patients considered for surgery with curative intent should receive routine brain imaging, regardless of clinical stage. Based on the frequency of adventitious diseases detected during preoperative testing, the present study indicates that preoperative brain MRI may help to detect other diseases posing a highly significant surgical risk. These include cerebral infarction, carotid artery stenosis and aneurysm.

Because recurrence is within skeletal structures in about 10% of lung cancer patients surgically treated in stages I and II [27], skeletal complications from bone metastases present a major challenge to disease management [28]. In a meta-analysis, both ^18^F-FDG PET and PET/CT were better for diagnosing bone metastasis from lung cancer than whole body MRI or BS because of their higher diagnostic values (sensitivity, specificity and diagnostic odd ratios) [29]. Although the isolated costs of standard staging using CT and BS are lower than the cost of PET/CT [30], given that the PET/CT strategy will enable diagnosis of more patients with advanced lung cancer, help prevent inappropriate surgery and shorten the length of hospital stays in connection with initial staging, the costs of the two staging strategies will likely not significantly differ. PET can replace BS for the evaluation of distant metastasis [31], as no diseases other than lumbar spondylosis were detected with BS in the present study.

A key aspect in lung cancer is multidisciplinary management in which there is a close collaboration between several medical specialties. Lung cancer care must only be carried out in lung cancer units or centres that have a core multidisciplinary team (MDT) and an extended team of health professionals [32]. Radiologists are involved in the early cancer detection, diagnosis, staging and play critical roles in the MDT. Since incidental findings of other diseases were frequently seen even in early lung cancer, the combination of various imaging modalities should be used by them to detect tumor characteristics, determine the clinical stage, and assist in assist in planning surgical therapy. The preoperative imaging combination will reveal the presence of adventitious diseases with the potential to increase surgical risks and worsen patient outcomes, especially in elderly patients.

This study has several limitations. First, there was variation with respect to the staging management, the radiologists, and the recommendations for further investigation of incidental findings. Consequently, some potentially significant incidental findings did not have subsequent imaging or clinical follow-up. Second, only 71.1% of patients received PET/CT in the present study. ^18^F-FDG PET and PET/CT are generally recognized as gold-standard imaging for evaluating lung cancer stage before surgery, but they are not readily available everywhere in all countries.

## Conclusions

In summary, our results indicate that there is no real need to check for distant metastasis in clinical T1N0 GGN patients during initial staging for lung cancer in concordance with existing clinical guidelines. However, with various imaging modalities there were incidental findings of other diseases with the potential of increase surgical risks and worsen patient outcomes, especially in elderly patients. Although there is not enough evidence, in certain cases, if clinically feasible, it could be considered to complement staging with whole-body procedures, such as ^18^F-FDG PET/CT etc.

### Electronic supplementary material

Below is the link to the electronic supplementary material.

Supplementary Material 1

## Data Availability

The datasets used and/or analyzed during the current study are available from the corresponding author on reasonable request.

## Abbreviations

NSCLC: non-small cell lung cancer
GGN: ground-grass nodule
BS: bone scintigraphy
ST: solid tumors
SE: spin-echo
MDP: methylene diphosphonate
HDMP: hydroxy methylene diphosphonate
UICC: the Union Internationale Contre le Cancer
OS: overall survival
RFS: relapse-free survival
DFS: disease-free survival
TIA: transient ischemic attack
EGFR: epidermal growth factor receptor
