# Supplementary material for: Does clinical T1N0 GGN really require checking for distant metastasis during initial staging for lung cancer?
Source: Cancer Imaging. 2024 Jun 3;24:69. doi: 10.1186/s40644-024-00714-7 (PMC11149246; doi:10.1186/s40644-024-00714-7)
Supplement: Supplementary file 1 — Supplementary Material 1 [file 40644_2024_714_MOESM1_ESM.docx]

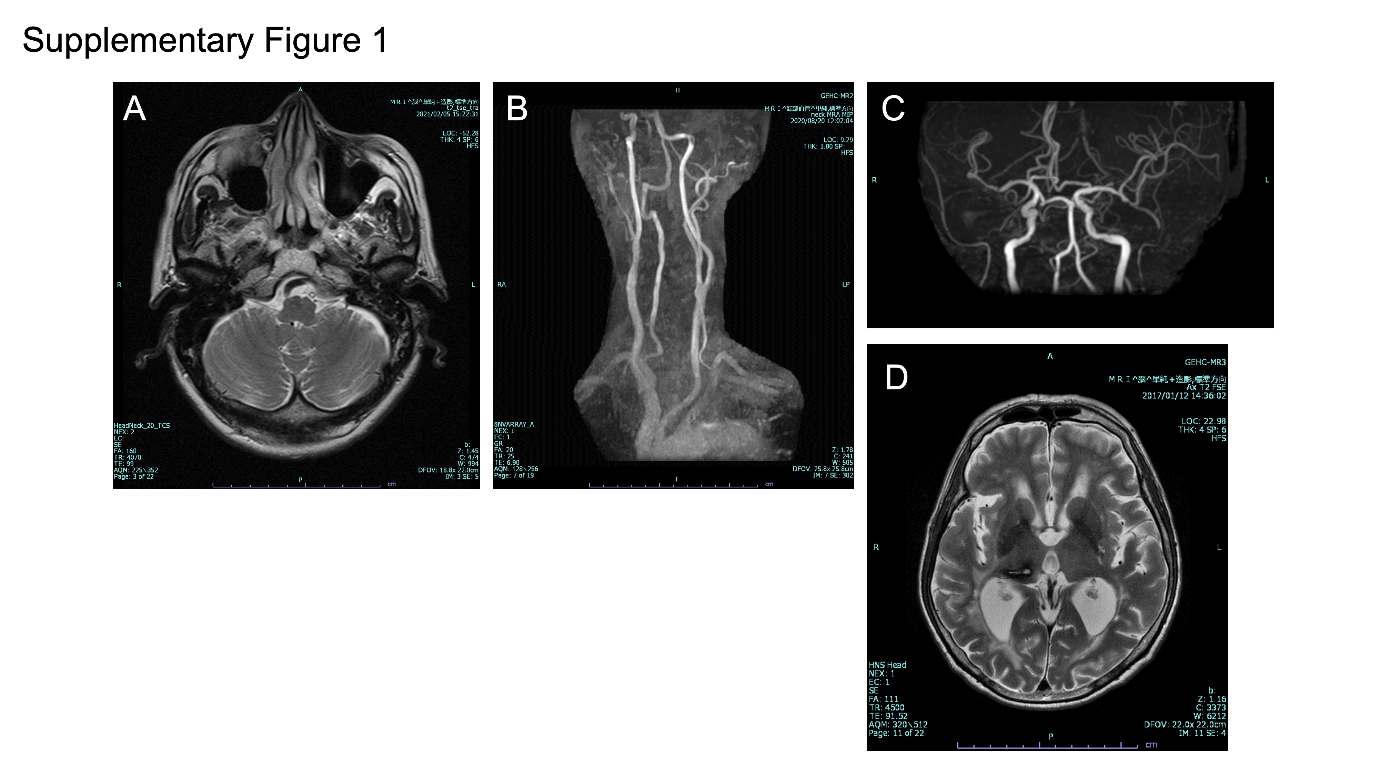


**Supplementary Figure 1.**

*Images of incidental findings considered to be high surgical risk candidates.*

(A) **Case 3** (67-year-old male with left S9 GGN): Vertebral artery occlusion was detected on MRI. The distal left vertebral artery was hypoplastic. This artery showed high signal on T1-weighted image (WI), and the flow void was unclear on T2WI. Case 3 remained asymptomatic.

(B) **Case 6** (78-year-old male with right S8/9 GGN): Stenosis at the origin of the left internal carotid artery was detected on MRI and MRA. High signal carotid plaques were observed on T1WI. Case 6 remained asymptomatic.

(C) **Case 13** (73-year-old male with right S8 solid squamous cell carcinoma): Right internal carotid artery stenosis and right middle cerebral artery-M1 stenosis were detected on MRI. The carotid artery wall showed slight high signal on fat-suppressed T1WI. Case 13 remained asymptomatic.

(D) **Case 19** (68-year-old male with right S6 solid squamous cell carcinoma): Old cerebral infarction following thalamic infarction/hemorrhage was detected on MRI. The thalamic hemorrhage had reduced without intervention. Case 19 had no paralysis as the aftereffects of infarction/hemorrhage.
